# Relationship between lower limb torsion and coronal morphologies of the femur and tibia in patients with medial knee osteoarthritis

**DOI:** 10.1186/s12891-020-03286-2

**Published:** 2020-04-17

**Authors:** Shuntaro Nejima, Yasushi Akamatsu, Hideo Kobayashi, Masaki Tsuji, Shota Mitsuhashi, Takahiro Sasaki, Ken Kumagai, Yutaka Inaba

**Affiliations:** Department of Orthopaedic Surgery, Yokohama City University School of Medicine, 3-9 Fukuura, Kanazawa-ku, Yokohama, 236-0004 Japan

**Keywords:** Femoral and tibial torsions, Hip-knee-ankle angle, Knee osteoarthritis, Lateral distal femoral angle, Medial proximal tibial angle

## Abstract

**Background:**

To investigate the relationship between femoral or tibial torsion and hip-knee-ankle angle (HKA), mechanical lateral distal femoral angle (mLDFA), or mechanical medial proximal tibial angle (mMPTA) in patients with medial knee osteoarthritis (OA).

**Methods:**

A total of 75 knees were enrolled. Femoral and tibial torsions were measured by superimposing the axial planes of computed tomography images. The relationship between femoral or tibial torsion and HKA, mLDFA, or mMPTA on radiographs was examined.

**Results:**

The mean femoral torsion was 12.2 ± 8.5° internally; femoral internal and external torsions were observed in 70 and 5 knees, respectively. The mean tibial external torsion was 18.0 ± 7.4° externally; tibial external torsion was observed in all 75 knees. Femoral internal and tibial external torsions increased with lower mMPTA (*r* = 0.33, *P* = 0.003; *r* = − 0.32, *P* = 0.005, respectively) but were not related to HKA or mLDFA.

**Conclusion:**

Femoral and tibial torsions were correlated with varus inclination of the proximal tibia in patients with medial knee OA.

## Background

Femoral and tibial torsions can be measured using computed tomography (CT) [1–25] and the values of patients with knee osteoarthritis (OA) were reported [2, 3, 6, 12, 14, 16–18, 21–24, 26, 27]. The mean femoral internal and tibial external torsions evaluated by CT were 15.7° to 19.7° (range, − 6° to 42°) and 11.3° to 27.7° (range, − 12° to 34.8°) in medial knee OA, respectively [3, 12, 21–24]. The range of femoral and tibial torsions was wide and factors related to both torsions remain unclear. It is important to evaluate femoral and tibial torsions, because the relationship between these values and deformity of knee OA was reported [2, 22, 23]. However, CT images are not always obtained in clinical practice.

Tibial external torsion decreased with increasing varus inclination of medial tibial plateau in medial knee OA [22, 23]. As lower limb alignment changed from valgus to varus, tibial external torsion decreased in medial and lateral knee OA [2]. The only relationship between femoral or tibial torsions, and lower limb alignment or the proximal medial tibial deformity in knee OA has been investigated. If factors related to the femoral and tibial torsions were revealed, the values of femoral and tibial torsions will be predictable without CT images. Lower limb alignment is defined by the hip-knee-ankle angle (HKA), which is measured on long leg radiographs [28]. Of the anatomical elements of lower limb alignment, the present study focused on varus deformity of the distal femur and proximal tibia and investigated the relationship with femoral and tibial torsions. It was hypothesized that femoral and tibial torsions were related to the coronal deformity of the femur and tibia, respectively. The present study aimed to investigate the relationship between femoral or tibial torsion, and the coronal deformity of the femur and tibia in patients with medial knee OA.

## Methods

A total of 57 female patients (75 knees) scheduled to undergo open wedge high tibial osteotomy (HTO) as treatment for medial knee OA from June 2011 to June 2016 were enrolled. The definition of knee OA was Kellgren-Laurence grade ≥ 2 on anteroposterior standing radiographs. Radiographs and CT images were preoperatively obtained for the purpose of the surgery. Patients who had osteonecrosis of the knee, had hip or ankle OA, or underwent previous lower limb surgery were excluded. The mean knee flexion angle was 127.8 ± 11.6° (range, 100°–145°) and extension angle was − 3.4 ± 5.1° (range, − 20°–0°).

### Measurements of lower limb alignment and coronal morphologies of the distal femur and proximal tibia

**Fig. 1:**
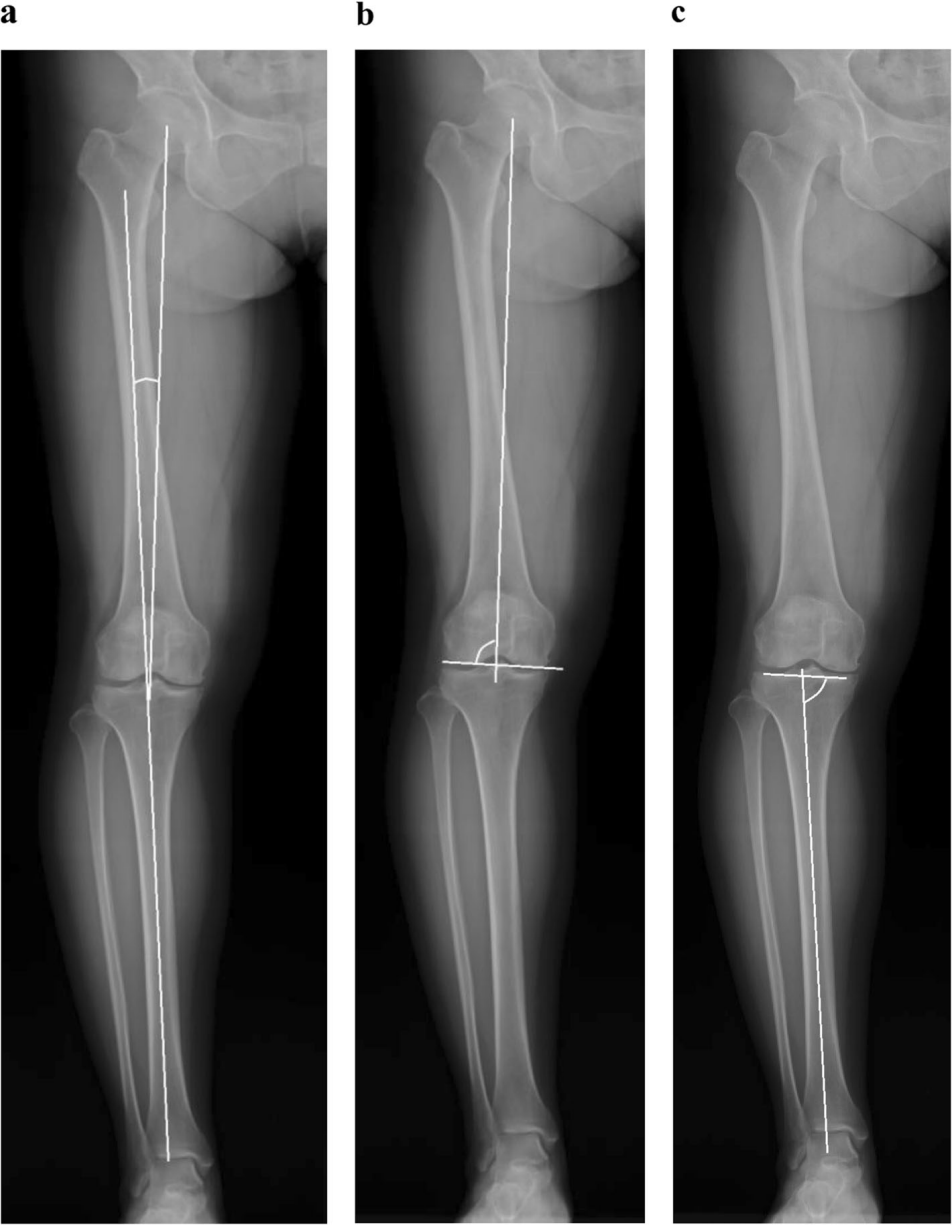
Measurements of hip-knee-ankle angle (HKA) (**a**), mechanical lateral distal femoral angle (mLDFA) (**b**), and mechanical medial proximal tibial angle (mMPTA) (**c**)

Anteroposterior whole-leg radiographs were obtained in one-leg standing position, with the knee joint maintained in extension. The lower limbs were positioned so that the patella faced forward and the X-ray beam centered on the knee. The radiographs obtained using a Fuji computed radiography system were projected, and HKA, mechanical lateral distal femoral angle (mLDFA), and mechanical medial proximal tibial angle (mMPTA) were measured using Fujifilm OP-A software (Fujifilm Co. Ltd., Tokyo, Japan) (Fig. 1a–c). HKA was defined as the angle between the mechanical axes of the femur and tibia; a positive value signified valgus alignment. Furthermore, mLDFA was defined as the lateral angle between the mechanical axis of the femur and the tangent to the femoral condyles, and mMPTA was defined as the medial angle between the mechanical axis of the tibia and the joint line of the proximal tibia. The mLDFA and mMPTA represent the coronal deformity of the femur and tibia, respectively. Normal values for each of the radiological parameters reported in literature are 85°–90° for mLDFA and mMPTA [29].

### Measurement of femoral torsion

With patients lying supine, whole-leg CT images were acquired using 1.5-mm-thick slices obtained on a SOMATOM Sensation 16 CT scanner (Siemens, Munich, Germany).

**Fig. 2:**
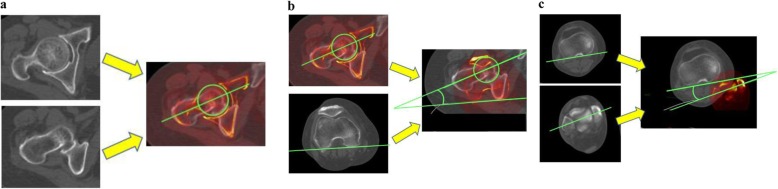
**a** The proximal femoral torsional axis was assessed by superimposing two axial planes of the center of the femoral head and the central axis of the femoral neck. **b.** Measurement of femoral torsion. Femoral torsion was defined as the angle between the proximal and distal femoral torsional axes. **c.** Measurement of tibial torsion. Tibial torsion was defined as the angle between the proximal and distal tibial torsional axes

Femoral and tibial torsions were measured using OsiriX software (Pixmeo, Genova, Switzerland), which enabled superimposition of axial planes of CT images. Femoral torsion was assessed using the method described by Reikerås et al. [20]. We initially selected axial planes of the center of the femoral head and the central axis of the femoral neck, and subsequently superimposed these planes. The proximal femoral torsional axis was defined as the line through the center of the femoral head and the central axis of the femoral neck (Fig. 2a). The distal femoral torsional axis was the line connecting most posterior edge of the femoral condyles. Femoral torsion was defined as the angle between the proximal and distal femoral torsional axes (Fig. 2b). If the distal torsional axis was external relative to the proximal torsional axis, the torsion angle was recorded as a positive value.

### Measurement of tibial torsion

Tibial torsion, which was assessed using the bimalleolar method [15], was defined as the angle between the proximal and distal tibial torsional axes. The proximal tibial torsional axis was defined as the line tangent to the posterior contour of the proximal tibia above the proximal end of the fibula. The distal tibial torsional axis was defined as the line through the midpoints of the articular surfaces of the medial and lateral malleoli (Fig. 2c). If the distal torsional axis was external relative to the proximal torsional axis, the torsion angle was recorded as a positive value. All parameters on radiographs and CT images were measured by two experienced orthopaedic surgeons (SN, YA).

### Statistical analysis

Data were expressed as mean ± standard deviation and normal distribution was confirmed using the Shapiro–Wilk test. Simple linear regression (Pearson correlation coefficient) was used to examine the relationship between femoral or tibial torsion, and HKA, mLDFA or mMPTA, and the relationship between femoral and tibial torsions. All statistical analyses were performed using IBM SPSS for Windows version 21.0 (IBM Corporation, Armonk, NY, USA). *P* values < 0.05 were considered statistically significant. A power analysis was performed on correlations (*r* = 0.5, significance level = 0.05, power = 0.80) using G*Power version 3.1.9.2 (Heinrich-Heine-Universität Düsseldorf, Germany). A priori power analysis resulted in a sample size of 26. To examine the reproducibility of each measurement, we randomly selected 20 knees, and intraclass correlation coefficient (ICC) (1, 1) and ICC (2, 1) were calculated for intra- and inter-observer reliabilities, respectively. The scoring system of Landis and Koch [30] was used to analyze the results—that is, almost perfect, > 0.81; substantial, 0.61–0.80; moderate, 0.41–0.60; fair, 0.21–0.40; and slight, 0.0–0.20.

## Results

**Fig. 3:**
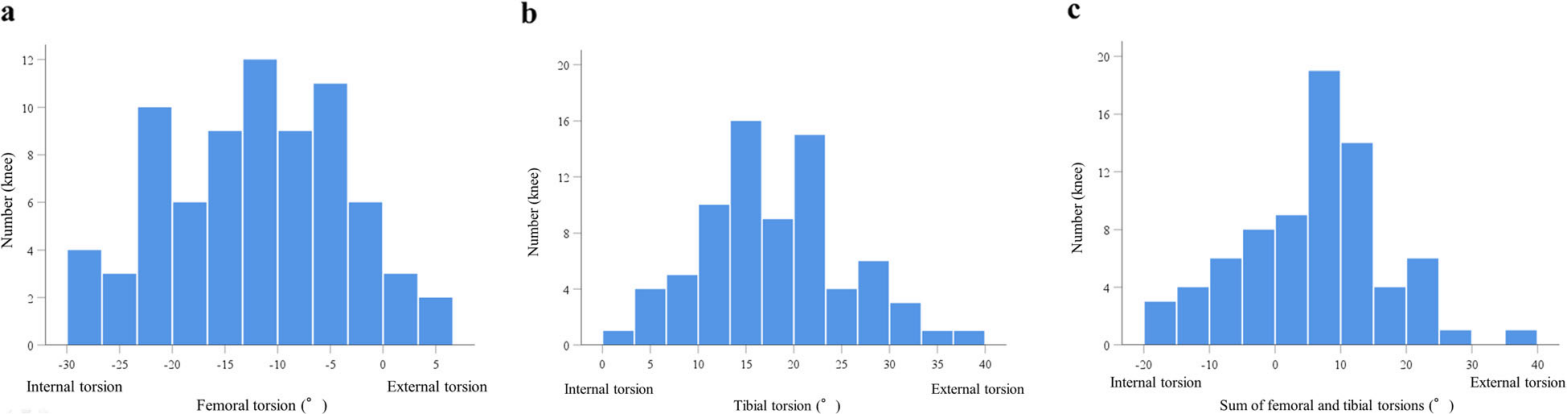
**a.** Histogram of femoral torsion. **b.** Histogram of tibial torsion. **c.** Histogram of sum of femoral and tibial torsions

**Fig. 4:**
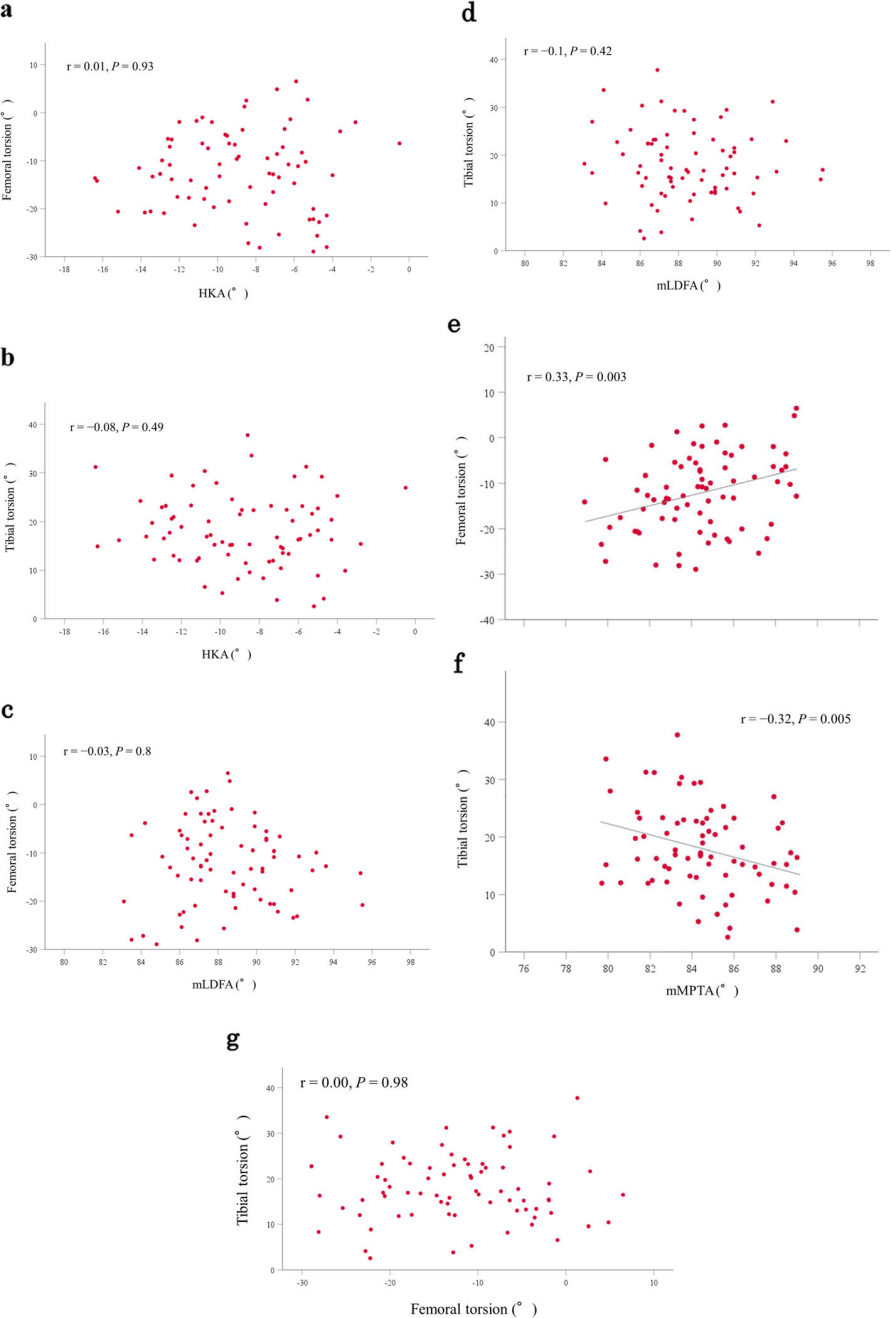
**a.** Relationship between hip-knee-ankle angle (HKA) and femoral torsion. **b.** Relationship between hip-knee-ankle angle (HKA) and tibial torsion. **c.** Relationship between mechanical lateral distal femoral angle (mLDFA) and femoral torsion. **d.** Relationship between mechanical lateral distal femoral angle (mLDFA) and tibial torsion. **e.** Relationship between mechanical medial proximal tibial angle (mMPTA) and femoral torsion. Femoral torsion increased with higher mMPTA. **f.** Relationship between mechanical medial proximal tibial angle (mMPTA) and tibial torsion. Tibial torsion increased with lower mMPTA. **g.** Relationship between femoral and tibial torsions

The demographic data are shown in Table 1, whereas the radiographic and CT data are summarized in Table 2. The mean femoral internal torsion was 12.2 ± 8.5°; femoral internal and external torsions were observed in 70 and 5 knees, respectively (Fig. 3a). The mean tibial external torsion was 18.0 ± 7.4°; tibial external torsion was observed in all 75 knees (Fig. 3b). The mean sum of femoral and tibial torsions was 5.9 ± 11.2° (Fig. 3c). Femoral internal and tibial external torsions increased with lower mMPTA (femoral internal torsion: *r* = 0.33, *P* = 0.003; tibial external torsion: *r* = − 0.32, *P* = 0.005). No relationship was observed between both torsions and HKA or mLDFA (Fig. 4a-f, Table 3). Femoral torsion was not related to tibial torsion (*r* = 0.00, *P* = 0.98 [Fig. 4g]). The intra- and inter-observer ICC values were 0.94 (95% CI 0.85–0.97) and 0.92 (0.76–0.97) for femoral torsion, 0.92 (0.82–0.97) and 0.91 (0.80–0.97) for tibial torsion, 0.99 (0.97–0.99) and 0.98 (0.84–0.99) for HKA, 0.89 (0.75–0.96) and 0.89 (0.75–0.95) for mLDFA, and 0.89 (0.74–0.95) and 0.81 (0.58–0.92) for mMPTA, respectively. All measured intra- and inter-observer ICCs were almost perfect according to the scoring system of Landis and Koch [30].

**Table 1:** Patient demographic characteristics

Knees, n	75
Age, yr	64.9 ± 7.7 (46–78).
Height, cm	154.8 ± 5.4 (138.5–172.1)
Weight, kg	60.8 ± 9 (43.8–85)
Body mass index	25.4 ± 3.6 (19–33.9)
Side, left/right	37/38
Kellgren-Laurence grade 3/4	37/38
Ahlbӓck grade 1/2/3	58/12/5

Data are presented mean ± standard deviation with range in the parenthesis

**Table 2:** Radiographic and CT data

HKA	−8.8 ± 3.4° (− 0.5°−− 16.4°)
mLDFA	88.4 ± 2.6° (83.1°–95.5°)
mMPTA	84.4 ± 2.5° (78.9°–89.0°)
Femoral internal torsion	12.2 ± 8.5° (− 6.5°–28.9°)
Tibial external torsion	18.0 ± 7.4° (2.6°–37.8°)
Sum of femoral and tibial torsions	5.9 ± 11.2° (− 19.8°–39.0°)

Data are presented mean ± standard deviation with range in the parenthesis. The mLDFA, mechanical lateral distal femoral angle; mMPTA, mechanical medial proximal tibial angle; HKA, hip-knee-ankle angle. A positive value means valgus alignment

**Table 3:** Relationship between femoral and tibial torsions and HKA, mLDFA, and mMPTA

	Femoral torsion	*P*	Tibial torsion	*P*
HKA	*r* = 0.01	0.93	*r* = − 0.08	0.49
mLDFA	*r* = − 0.03	0.8	*r* = − 0.1	0.42
mMPTA	*r* = 0.33	0.003	*r* = − 0.32	0.005

HKA, hip-knee-ankle angle; mLDFA, mechanical lateral distal femoral angle; mMPTA, mechanical medial proximal tibial angle

## Discussion

The important findings of the present study were that femoral internal and tibial external torsions increased with lower mMPTA, but were not related to HKA or mLDFA in patients with medial knee OA. In addition, femoral internal and tibial external torsions neutralized each other, but were not correlated.

With respect to tibial torsion and loading distribution on the tibia, Yazdi et al. [31] investigated the relationship between tibial torsion and medial and lateral compartment contact pressure in the knee joint using cadaveric specimens. They reported that a 15° external change in tibial torsion decreased the medial compartment contact pressure and increased the lateral compartment contact pressure. In contrast, a 15° internal change in tibial torsion increased the medial compartment contact pressure and decreased the lateral compartment contact pressure. In the present study, tibial external torsion increased with lower mMPTA. This might be a compensation mechanism for the varus deformity of the tibia.

Interestingly, femoral internal torsion increased with lower mMPTA in the present study. We speculate that the relationship between femoral internal torsion and lower mMPTA might have restored the change in joint line obliquity in continuous flexed knee position. In the case with femoral internal torsion and normal mMPTA, the tibial axis in the coronal plane will change to valgus if the femur is fixed and the knee is flexed. Internal torsion of the posterior medial femoral condyle has a tendency to move the medial joint line down in flexed position. Therefore, the relationship between femoral internal torsion and lower mMPTA shows a physiological interaction to adjust the change in joint line obliquity and coronal tibial alignment in flexed position. Moreover, femoral internal torsion and lower mMPTA might enable the joint line to be parallel to the ground in continuous flexed position.

Femoral internal torsion was correlated with tibial external torsion in normal cadaveric specimens in a previous study [32]. Meanwhile, femoral internal torsion was not correlated with tibial external torsion in the present study. Duparc et al. [3] evaluated the sum of femoral and tibial torsions as the index of cumulative torsions (ICT). They reported that ICT was highly variable and there were three morphotypes of ICT in patients with medial knee OA. In accordance with their result, ICT was highly variable in the present study. This might lead to no correlation between femoral and tibial torsions in the present study. On the other hand, mMPTA was correlated with both femoral and tibial torsions in the present study. The clinical relevance of this study is that the values of femoral and tibial torsions can be predictable from the varus inclination of the proximal tibia on radiographs in patients with medial knee OA. Although correlation coefficients in this study were not high and further studies are needed, this study may be helpful for the prediction of femoral and tibial torsions on radiographs in future.

The present study has a few limitations. First, the present results were obtained from Japanese female patients. Whether these results can be generalized to male patients is unclear. We excluded male patients in consideration of significant sex difference in femoral torsion among subjects with medial knee OA [17]. However, the prevalence of knee OA was higher in female than in male in Japanese population [33]. Thus, the result of the present study can be applicable to many patients in Japan. Second, the extent of OA in patients in this study was classified as Ahlbӓck grade 1 to 3. Whether mMPTA is related to femoral and tibial torsions in a healthy population or in patients with more severe osteoarthritic knees is unknown. Third, the lower limb position on CT scan and flexion contracture may affect the measured value on CT images. However, the measurement of femoral and tibial torsions using CT was not affected by position when the femorotibial angle was less than 195° and flexion contracture was less than 20° [21]. Every patient in the present study underwent open wedge HTO, and the range of knee extension angle was − 20°–0°. Our indications for open wedge HTO include femorotibial angle less than 185°. Therefore, the measurement of femoral and tibial torsions was less influenced by the lower limb position and flexion contracture. Fourth, femoral and tibial torsions were measured in the supine position, whereas HKA, mLDFA, and mMPTA were measured on standing radiographs. This is a retrospective study and one-leg standing radiographs were taken for the surgical planning. Anteroposterior standing radiographs could increase joint line convergence angle and affect HKA, compared with supine radiographs. However, mLDFA and mMPTA might be less affected by anteroposterior standing radiographs.

## Conclusion

Femoral and tibial torsions were correlated with varus inclination of the proximal tibia in patients with medial knee OA.

## Data Availability

The datasets used and/or analyzed during the current study available from the corresponding author on reasonable request.

## Abbreviations

CT: Computed tomography
OA: Osteoarthritis
HKA: Hip-knee-ankle angle
mLDFA: mechanical Lateral distal femoral angle
mMPTA: mechanical Medial proximal tibial angle
HTO: High tibial osteotomy
ICCs: Intraclass correlation coefficients
ICT: Index of cumulative torsions
